# Analysis of water level variation of lakes and reservoirs in Xinjiang, China using ICESat laser altimetry data (2003–2009)

**DOI:** 10.1371/journal.pone.0183800

**Published:** 2017-09-05

**Authors:** Zhaoxia Ye, Hongxing Liu, Yaning Chen, Song Shu, Qiusheng Wu, Shujie Wang

**Affiliations:** 1 State Key Laboratory of Desert and Oasis Ecology, Xinjiang Institute of Ecology and Geography, Chinese Academy of Sciences, Urumqi, Xinjiang, China; 2 Department of Geography and Geographic Information Science, University of Cincinnati, Cincinnati, Ohio, United States of America; 3 Department of Geography, Binghamton University, State University of New York, Binghamton, United States of America; Bristol University/Remote Sensing Solutions Inc., UNITED STATES

## Abstract

This study utilizes ICESat Release 33 GLA14 data to analyse water level variation of Xinjiang’s lakes and reservoirs from 2003 to 2009. By using Landsat images, lakes and reservoirs with area larger than 1 km^2^ are numerically delineated with a software tool. Based on ICESat observations, we analyse the characteristics of water level variation in different geographic environments, as well as investigate the reasons for the variation. Results indicate that climatic warming contributes to rising water levels in lakes in mountainous areas, especially for lakes that are recharged by snow and glacial melting. For lakes in oases, the water levels are affected jointly by human activity and climate change, while the water levels of reservoirs are mainly affected by human activity. Comparing the annual average rates of water levels, those of lakes are higher than those of reservoirs in oasis areas. The main reasons for the decreasing water levels in desert regions are the reduction of recharged runoff and high evaporation. By analysing the variation of water levels and water volume in different geologic environments, it is found that water level and volume increased in mountainous regions, and decreased in oasis regions and desert regions. Finding also demonstrate that decreasing volume is greater than increasing volume, which results in decreasing total volume of Xinjiang lakes and reservoirs.

## Introduction

It was reported that total glacier areas in China have decreased by approximately 5.5% over the past 45 years [1]. These processes could greatly affect water resource distribution [2]. In the northwestern arid region, mountain snowmelt is an important source of water and is critical to local inhabitants’ livelihood and socio-economic development [3]. Lakes, as primary water resources, play important roles in water supply and regulation [4].

Over the past 50 years, Li et al. [5] showed that temperatures and precipitation in the arid region of northwest China demonstrate significant increasing trends (P<0.01), with rates of 0.343°C per 10 years and 6.07 mm per 10 years, respectively. This is substantially higher than the global average (0.13°C per 10 years) [5]. In different environments, the rates of increase are different [6]. Variations of temperature and precipitation influence the snowmelt period, as well as runoff in the mountainous areas in northwest China [7].

Xinjiang is located in the interior of the Eurasian continent and far from the sea. Its unique location and landform pattern of mountain-basin structure formed the unique climatic characteristics of Xinjiang. There are wildly distributed desert and gobi in Xinjiang. In addition, many mountain-basin systems exist, constituted by staggered high mountains and basins, in which a large number of oasis systems developed. Mountain systems, oasis systems and desert systems interact to form a large number of mountain-oasis-desert systems [8].

The water level variation of lakes can reflect water resource changes more accurately than area variation of lakes, which is a comprehensive indication of the impact of climate change and human activity on regional water resources. For example, for some lakes, due to basin geometry, increasing or decreasing water volume does not lead to significant changes in the lake area, while water level changes would be more obvious. In other words, water level changes are more sensitive to water volume changes. As natural resources, lakes accommodate rain, as well as extra water from rivers when it rains. Lakes also supply previously stored water to rivers and surrounding soil when the climate becomes dry. Artificial reservoirs focus more on function. They are formed by the construction of a dam in a narrow mouth of a ravine or river. Reservoirs play important roles in flood control, water storage for irrigation, water supply, power generation, fishing, etc. Declining water levels of lakes and reservoirs will produce a series of environmental and socio-economic problems, such as wetland area shrinkage, species diversity reduction, water quality deterioration, fish production reduction, crop water shortage, etc. Keeping water levels of lakes and reservoirs at a reasonable level is critical for the purposes of environmental protection and social stability.

We can extract lake or reservoir boundaries by using traditional optical and microwave remote sensing. However, monitoring changes in surface water elevation is not an easy task. Typically, the traditional approach is to discern water levels through field measurements, and then plot the water level curves with time variations in order to obtain water level variations. However, not all lakes have field monitoring data, especially in underdeveloped regions or remote mountainous areas with harsh environments. Therefore, we cannot easily determine the water levels of lakes and reservoirs on large scale, which has made achieving a comprehensive understanding of water level variations difficult within the scope of Xinjiang. Considering the importance of water levels research and the difficulty of large scale monitoring of water levels, a technology is urgently needed that can be utilized to achieve a wide range of water level monitoring.

The Geoscience Laser Altimeter System (GLAS) on Ice, Cloud, and Elevation Satellite (ICESat), was launched successfully by the U.S.A. National Aeronautics and Space Administration (NASA) in 2003. The geographical coverage ranges from 86°N to 86°S. ICESat elevation data over water/flat surface in east Africa, southern Egypt and the U.S.A are examined by numerous studies, and have verified accuracy of better than 10 cm [9]. It is also demonstrated that the precision of the elevation estimates, measured over relatively flat sea ice, is 2 cm over 70 m laser footprints spaced at 172 m [10]. The ICESat/GLAS elevation dataset has been widely applied to measure the thickness of the polar ice caps, monitor the melting of snow and the ice shelf [11–13], monitor the sea ice freeboard [14–18], biomass estimates [19, 20], measure the water elevation variation of lakes [21], snow depth [22], etc.

ICESat/GLAS data were also used for studies on the water elevation of lakes in China in recent years [2, 9, 23, 24]. Many scholars have been concerned about large lakes with areas larger than 100 km^2^ throughout the nation [2], and especially in the Tibetan Plateau. For example, Song et al. [24] researched seasonal and abrupt changes in the water level of closed lakes on the Tibetan Plateau and implications for climate impacts. They found that most lakes in southern Tibet and the central plateau experienced relatively more negative lake-level shifts in cold seasons, partly due to a smaller snow melt water supply, but larger lake-level rises in warm seasons due to the higher precipitation that was influenced by warm-and-moist Indian Monsoon and East Asia Monsoon, compared with lakes in the Changtang Plateau and around the Kunlun Mountains. Zhang et al. [9] analysed water level change trends of 74 lakes on the Tibetan Plateau with ICESat footprints for 4–7 years. It was concluded that the recent increases in lake levels, supported accelerated glacier melting with global warming as the most likely cause. Zhu et al. [25] analysed the fluctuation of Lake Qinghai using multi-source remote sensing data (ICESate data and the Moderate Resolution Imaging Spectroradiometer product) and demonstrated that it was feasible to comprehensively monitor the fluctuation of large water bodies based entirely on remote sensing data.

For Xinjiang lakes, most studies have been limited to individual large lakes [26–32] or those lakes with more complete information and data [33–35]. Almost no extant literature exists about water level variations of lakes and reservoirs in Xinjiang. Xinjiang is sensitive to climate changes, which is reflected in water level variations. This study utilizes ICESat GLA14 data to analyse the water level variation of Xinjiang’s lakes and reservoirs from 2003 to 2009. By utilizing Landsat images, lakes and reservoirs with areas larger than 1 km^2^ are numerically delineated with a software tool. In order to elucidate the changes of water level of lakes under different environments and their influencing factors, we attempt to divide all lakes into mountainous lakes, oasis lakes, and desert lakes. Combined with geographical distribution characteristics, DEMs and Landsat images, we divided the region of Xinjiang into three types of regions. Regions higher than 1500m are mountain regions, lakes located in which are defined as mountainous lakes. For the remaining area, we classified oasis and desert regions by Landsat images. Specifically, lakes located in oasis and desert regions are defined as oasis lakes and desert lakes, respectively. The same classification system is used for reservoirs. We analysed the water level variations of lakes and reservoirs in different geologic environments and examined the corresponding reasons. The difference of water level variations between lakes and reservoirs in the same geologic environments was also analysed. This study can serve as a useful reference for water level study in remote regions, and provides decision-making guidance for macroeconomic regulations and control of water resources.

## Materials and methods

### Study area

Xinjiang is a typical semi-arid and arid area, located in northwest China. Its 1.66 million square kilometres represent approximately one-sixth of the total territory of the country. The region possesses a unique landscape, which is called “three mountains surrounding two basins”. From south to north, they are the Kunlun Mountains, Tarim Basin, Tianshan Mountains, Junggar Basin, and Altay Mountains. The Tianshan Mountains, divide Xinjiang into northern and southern parts. The annual mean temperature in the area is 10–15°C, and the annual precipitation is less than 150 mm, which is only 23% of the average level in China (630 mm). Because of the dry climate, evaporation in Xinjiang is very strong, with a mean annual pan evaporation between 1000 and 4500 mm, which is 500–1000 mm higher than other locations at the same latitude in China [36]. In Xinjiang, the mountains are runoff-forming areas due to the abundant precipitation. Oases are the areas of water resource consumption. Deserts are the dissipation areas of runoff.

### Data sources

**Fig 1:**
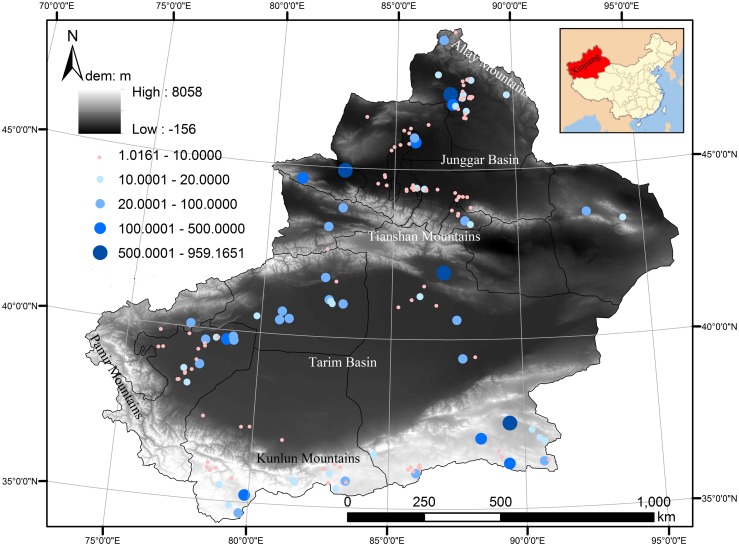
Area and distribution of lakes and reservoirs in Xinjiang, China.

We used Landsat 5 images of good quality between July and September 2006, which are downloaded from the United States Geological Survey (USGS) official website (https://earthexplorer.usgs.gov/). Good quality standard is cloud cover of less than 20%, and with a clear water boundary. The reasons that we chose image data in 2006 are as follows: 1) from July to September, 2006, the quality of the data is better and 2) regardless of from which year the data are taken during the period of 2003–2009, as long as good images are available, the study will not be adversely affected. During the extraction of water level, we then narrow the scope of water boundary to ensure that the water level data fall into the scope as much as possible. Even if the water level data do not fall into the water range, we will further eliminate outliers. All means are used to ensure that the final water level data are as close as possible to the real data. If there are no good images available for a region, we use images from other dates or other years as a substitution. Finally, a total of 45 images were collected, fully covering the water areas of Xinjiang. The resolution of Landsat 5 is 30 m. Using ArcGIS and shoreline extractor software [37], lakes and reservoirs with areas larger than 1 km^2^ were numerically delineated. A total of 155 lakes and reservoirs were extracted, and the map of the spatial distribution of lakes and reservoirs is presented in Fig 1. The border of China with administrative divisions is downloaded from the website: http://www.sbsm.gov.cn/. The Map Audit Number is GS(2016)1593. Table 1 shows the numbers and area of lakes and reservoirs in different administrative regions of Xinjiang, China. The total area is approximately 6672 km^2^. Among them, Bayingol is the region with the largest area of lakes and reservoirs, at 2743.90 km^2^, which accounts for 41% of the total area. Yili is the region with the largest number, comprising 47 lakes and reservoirs, which accounts for more than 30% of the total number. There are 10 lakes and one reservoir with areas larger than 100 km^2^. Of the total lakes and reservoirs, 45 are located in mountains, 103 are located in oases, 7 are located in deserts (Table 2). The area of lakes and reservoirs in mountains is approximately 2693.79 km^2^ (accounting for 40.4% of total area); area in oases is approximately 3197.18 km^2^ (accounting for 47.9% of total area); and area in deserts is approximately 781.02 km^2^ (accounting for 11.7% of total area).

**Table 1:** Quantity and area in different administrative regions of Xinjiang, China.

Region/City		Quantity	Area(km^2^)	Region/City	Quantity	Area(km^2^)
Urumqi		5	45.48	Kezilesukeerkezi	2	68.67
Karamay		5	66.45	Bortala	2	1019.42
Yili	Kuitun	0	0	Turpan	0	0
	Yining	3	91.39	Hami	2	49.51
	Altay	27	1258.10	Kashigar	21	349
	Tacheng	17	238.51	Hetian	23	419.67
Bayingolin		27	2743.90	Aksu	10	270.21
Changji		11	48.14	**Total**	**155**	**6672**

**Table 2:** Quantity and area in different geologic environments of Xinjiang, China.

Geologic environments	Quantity	Area (km^2^)	Area/total area
mountain	45	2693.79	40.4%
oasis	103	3197.18	47.9%
desert	7	781.02	11.7%
total	155	6672	100%

**Fig 2:**
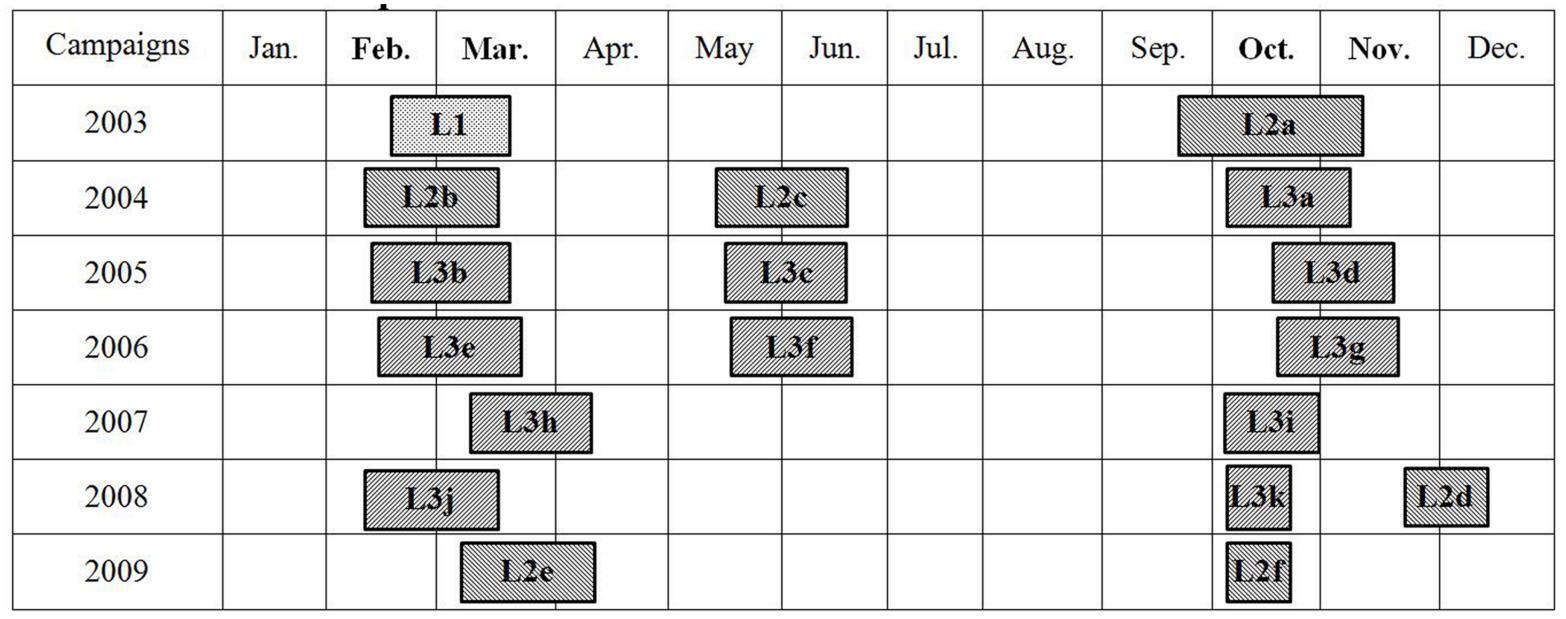
Observation period of ICESat/GLAS data.

The GLAS instrument provides a new, precise and global view of the vertical dimensions of the Earth’s surface. The altimeter range resolution is <3 cm for flat surfaces [38]. In this paper, ICESat GLA14 data crossing Xinjiang during 2003–2009 are downloaded from the National Snow and Ice Data Center (NSIDC). ICESat/GLAS data are taken two or three times a year from 2003 to 2009 and data from 19 campaigns (the duration of each campaign is approximately 33 days) have been obtained. The observation period of ICESat/GLAS data is shown in reference [2] (Fig 2). Unfortunately, there are no data on January, July to September, and December.

*In situ* gauge data of Bosten Lake water levels are collected from the Water Conservancy Bureau of Bayingolin Mongol Autonomous Prefecture. A traditional method of water gauge recording was adopted to monitor water levels. Monitoring frequency was twice a day, at 08:00 and 20:00.

### Data processing

Laser footprints were firstly extracted for each lake using the lake polygon obtained from Landsat images. Due to the seasonal and annual variation of lakes boundaries, and to ensure that the extracted footprints are within the lakes’ extent, a 200 m inward buffer was applied to all the lake polygons prior to extraction. In total, there were 56 lakes and reservoirs passed by ICESat/GLAS. After extraction, it was necessary to remove abnormal values of the extracted water level elevations. The elimination method is similar to the ones used in Wang et al. [39]. From the total 56 lakes and reservoirs, six with questionable data were excluded. Thus, a total of 50 are available (details in S1 Table).

## Results

### Validation of ICESat/GLAS data

**Fig 3:**
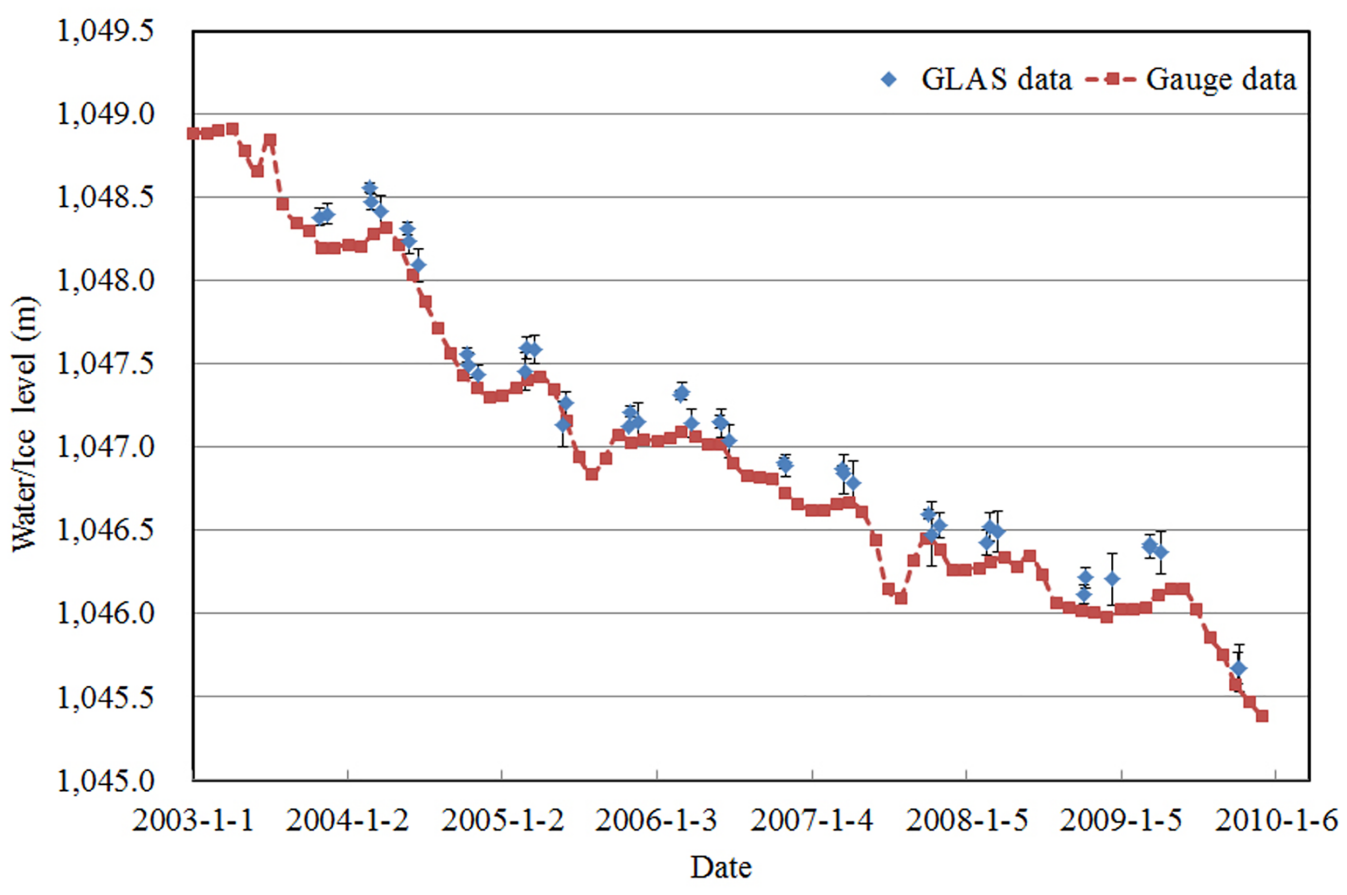
Water levels of Bosten Lake, derived from gauge and ICESat/GLAS data.

**Fig 4:**
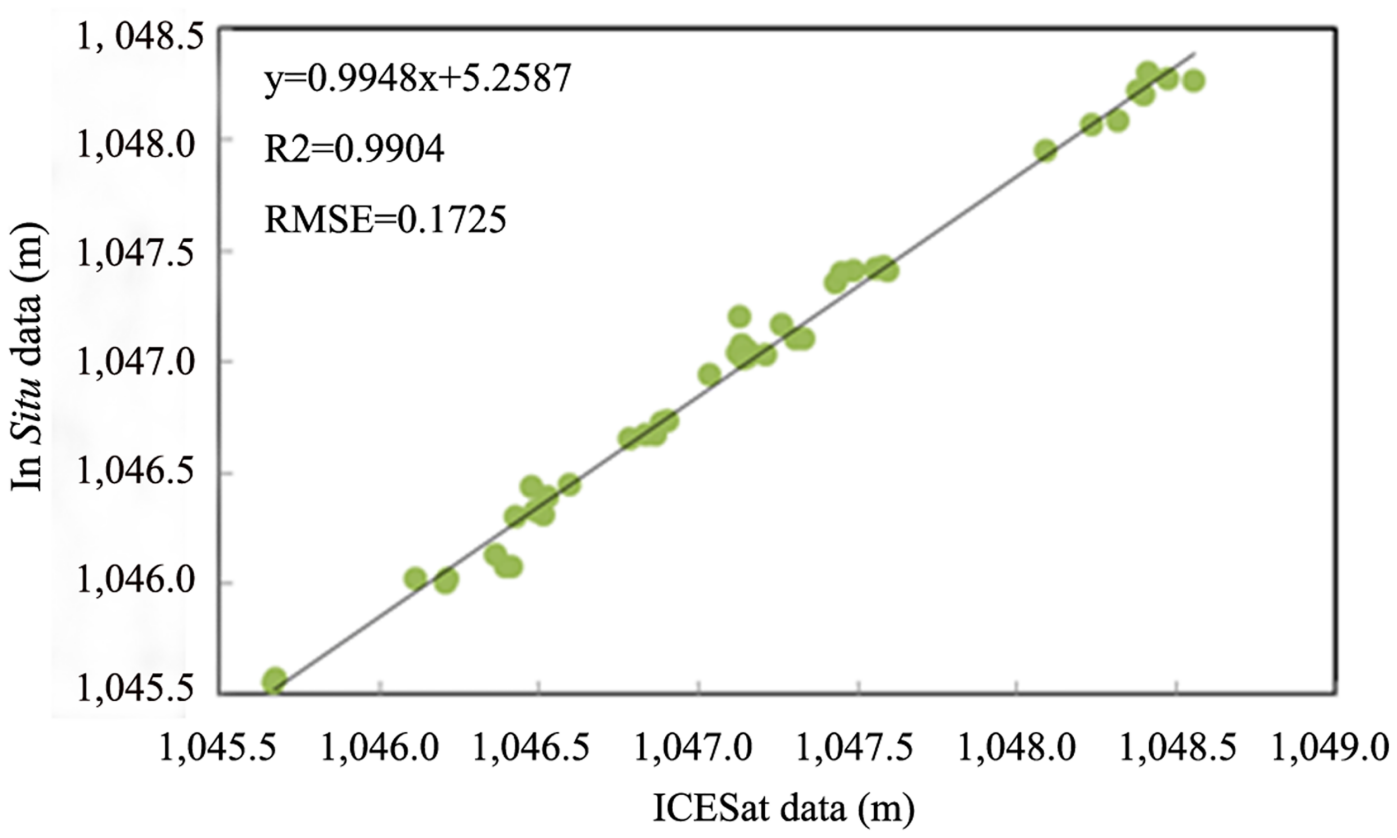
Relationship between GLAS data and interpolated gauge data of Bosten Lake.

Although the ICESat/GLAS data had a high resolution and a proven high level of accuracy in many research areas, it was still necessary to evaluate its data quality in the Xinjiang region. Bosten Lake, as the largest inland freshwater lake in China, was chosen as a validation site since we possess *in situ* gauge data on the first day of every month from 2003 to 2009. Bosten Lake (41°49´N–42°08´N, 86°41´E–87°27´E) [Wang et al. 1998] is located in the south of Xinjiang. The water level data from ICESat/GLAS and *in situ* gauge were plotted in Fig 3. It can be seen that the temporal trend is consistent between ICESat/GLAS and gauge data. The mean of ICEsat data and in situ data is 1047.12 m and 1046.97 m, respectively, the difference is 0.15 m, which is the system error between ICEsat data and in *situ* data. Moreover, they both have the same standard deviation, 0.756 m. To further quantitatively evaluate their consistency, we interpolated the gauge data to the GLAS data. The water level data from the in-situ gauge station observation data were validated using the ICESat/GLAS datasets. As the acquisition dates of these two data sources may be inconsistent, a linear interpolation method was adopted to estimate the data from the gauge station corresponding to the dates of ICESat/GLAS measurements. The linearly interpolated ICESat/GLAS measurements were then plotted with in-situ datasets, and the correlation coeff icient was estimated, as illustrated by Fig 4. Fig 4 showed good agreement between the variation of water levels from the gauge and GLAS data (R^2^ = 0.9904, RMSE = 0.1725).

### Seasonal variation of water level of Xinjiang lakes

**Fig 5:**
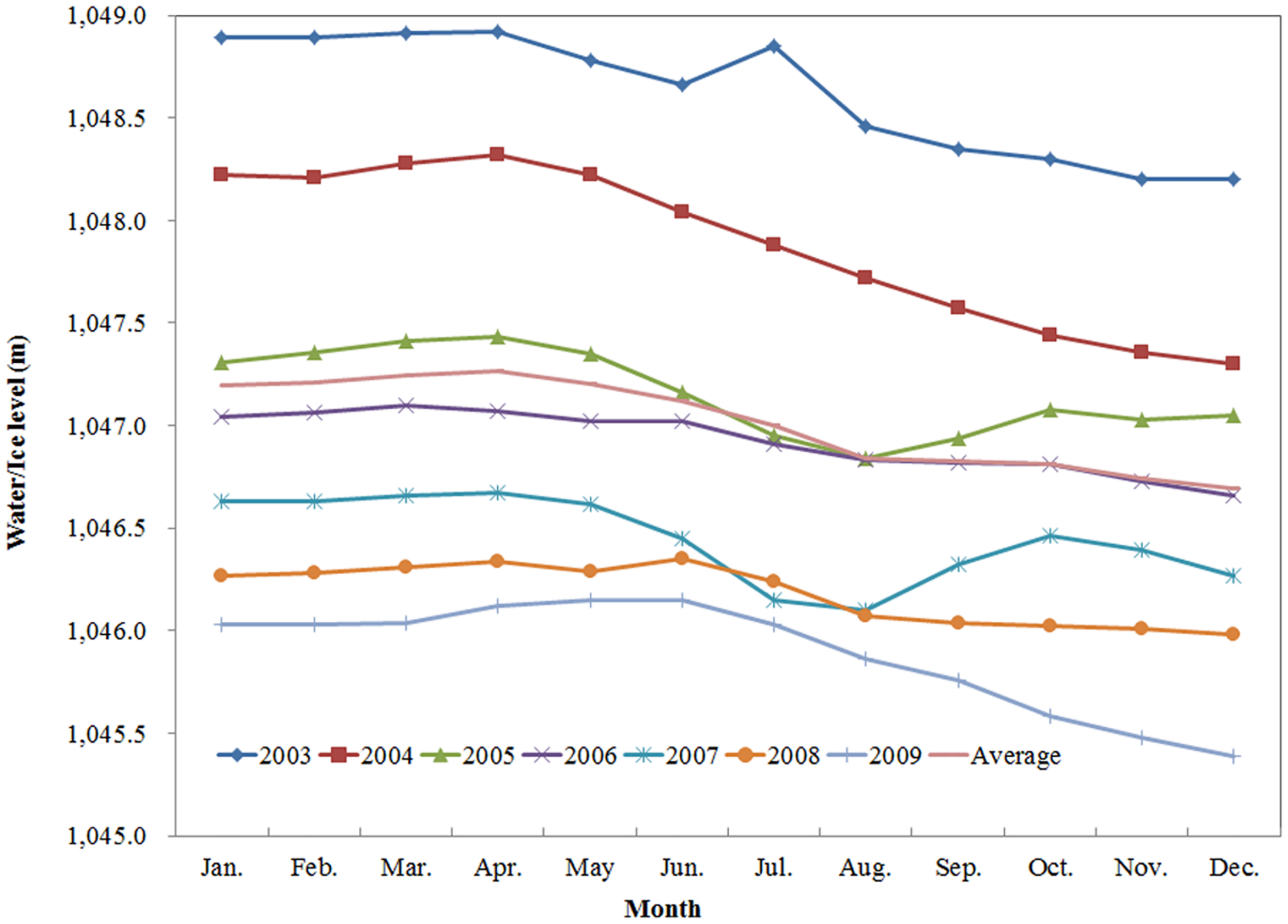
Monthly variation of water level in Bosten Lake by gauge data.

For Bosten Lake, Fig 3 shows obvious fluctuations every year. From Fig 5, however, water levels of each month in different years exhibited similar temporal distributions. The maximum water level usually occurred in April, and occasionally in March and June. The minimum water level occurred in December, and occasionally in August and November. Commonly, water surface elevation reached the lowest surface elevation in autumn after summer evaporation and irrigation, and reached the highest elevation in winter or spring after snow accumulation. The water surface annual elevation change can be as high as 1–2 m.

**Fig 6:**
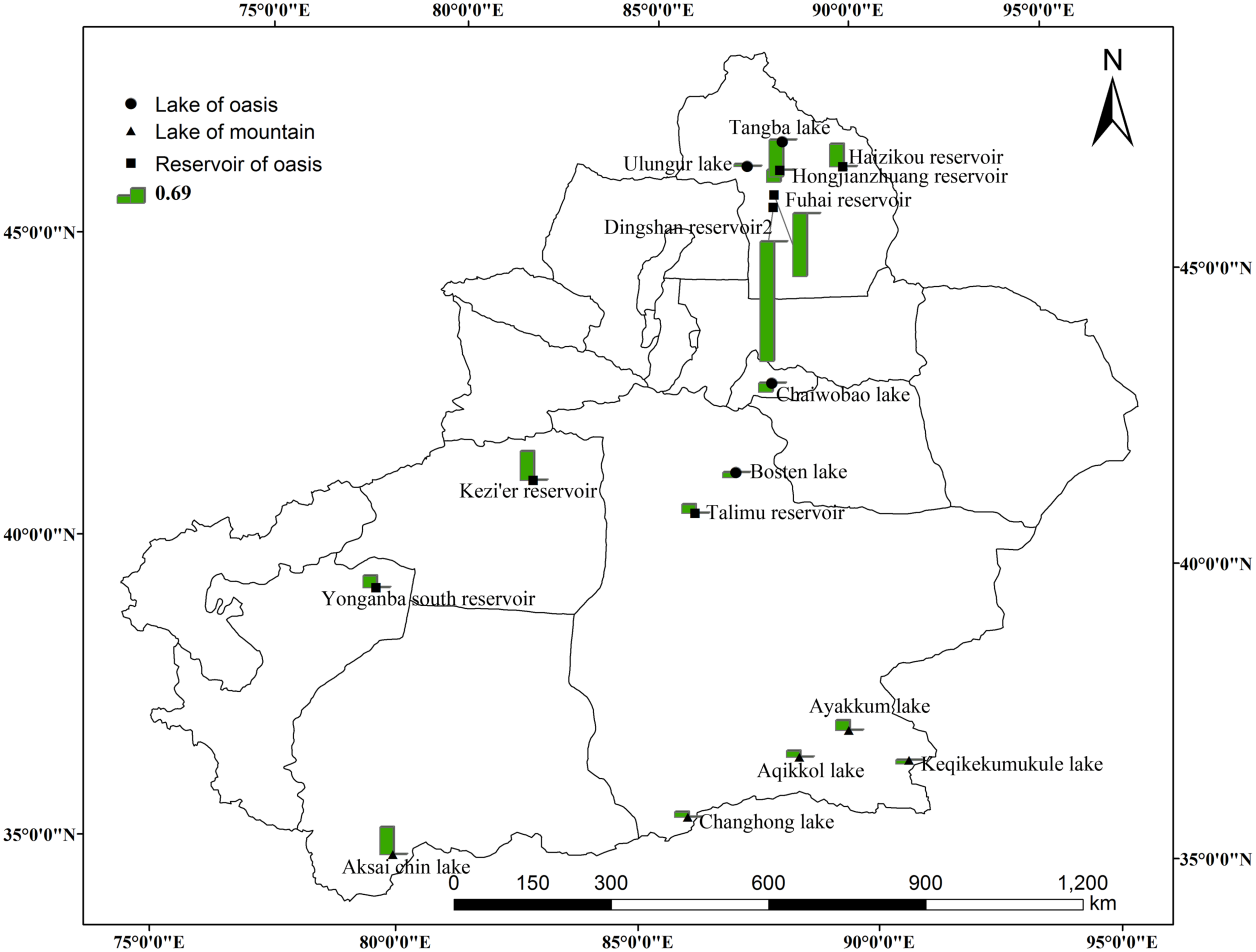
Spatial distribution of water level variation of Xinjiang lakes and reservoirs during the year. (Note: the histogram is the water level difference between autumn and spring. The column chart above the short line means an increase, and below the short line means a decrease).

Since there are little data in summer and no data in winter (Fig 2), we chose the difference between t autumn (October) and spring (March) to reflect the variation of lakes. For different lakes, the ICESat orbits passed over on different dates. Even for the same lakes, in different years, the ICESat orbits passed over on different dates. Therefore, this paper chose water level data in 2006 (a typical representative year) for space analysis of seasonal variation. The results showed that (Fig 6) most lake levels in the oasis areas decreased during the year. Specifically, the water level in spring was higher than that in autumn, in such lakes as Tangba Lake, Bosten Lake, and Chaiwopu Lake. However, most lake levels in the mountain areas increased during the year. Specifically, the water level in spring was lower than that in autumn, in such lakes as Aqikkol Lake, Ayakkum Lake, Aksai chin Lake and Changhong Lake. In addition, the reservoirs in the oasis areas have no specific rules, in that the number of reservoirs exhibiting rise and fall variation is almost the same, but the change value of the water level of reservoirs is higher than that of natural lakes. For reservoirs, the largest decrease of water level was 5.76m (Dingshan Reservoir2), and the smallest decrease of water level was 0.57m (Hongjianzhuang Reservoir). The largest increase was 1.39m (Kezi'er Reservoir), and the smallest increase was 0.41m (Talimu Reservoir). For lakes, the largest increase of water level was 1.31m (Aksai chin Lake), and the smallest increase was 0.1m (Ulungur Lake). Moreover, the largest decrease was 1.77m (Tangba Lake), and the smallest decrease was 0.18m (Keqikekumukule Lake). The difference of seasonal variation of water level reflects different factors. The oasis areas are water resource consumption areas and farming in Xinjiang is mainly distributed on the edge of the basin, which is characterized by irrigation agriculture. The water of lakes in the oasis areas is mainly used for agricultural irrigation or groundwater recharge. Summer is the season with the most irrigation water consumption, and the season with the strongest evaporation. After summer irrigation, evaporation and recharge, the water level of oasis lakes is lower in the fall. Mountainous lakes are affected mostly by climatic conditions, such as temperature, precipitation, and snow melt.

### Characteristics of water level variation in different geologic environments

The majority of observation periods are from February to March and from October to November. This is because rivers are most likely to flood in Xinjiang during the spring or summer [40], which leads to an increase of uncertainty about the water level variation of lakes. In addition, a large number of lakes will be frozen in the spring. As a result, data from October to November are chosen to calculate the annual average variation rate. We calculate the annual rate of water level variation in autumn by Eq (1).

$$Rate_{n,n+1}=\frac{H_{n+1}-H_{n}}{D_{n,n+1}}\times 365$$ (1)

where, *Rate*_*n*,*n*+1_ is the annual rate of water level variation between year n and n+1, unit: m/yr; *H*_*n*+1_ is the water elevation in year n+1 (we chose the elevation of one day between October to November to represent annual elevation, on the one hand, because there is much more data on October to November, on the other hand, the variations of water level tend to be stable, with weaker evaporation, less irrigation, and being in the freezing period), unit: m; *H*_*n*_ is the water elevation in year n, unit: m; and *D*_*n*,*n*+1_ are the days between two dates (we attempted to choose values of close dates of adjacent years). As a result, the annual average rate from 2003 to 2009 can be calculated by arithmetic average *Rate*_*n*,*n*+1_ s.

There are various changes in different lakes and reservoirs. In order to determine whether these variations depend on location, we categorized them into three types according to the special geologic setting of Xinjiang: lakes from mountains, lakes from oases and lakes from deserts. We then investigated whether there are regional characteristics of the water level changes.

**Fig 7:**
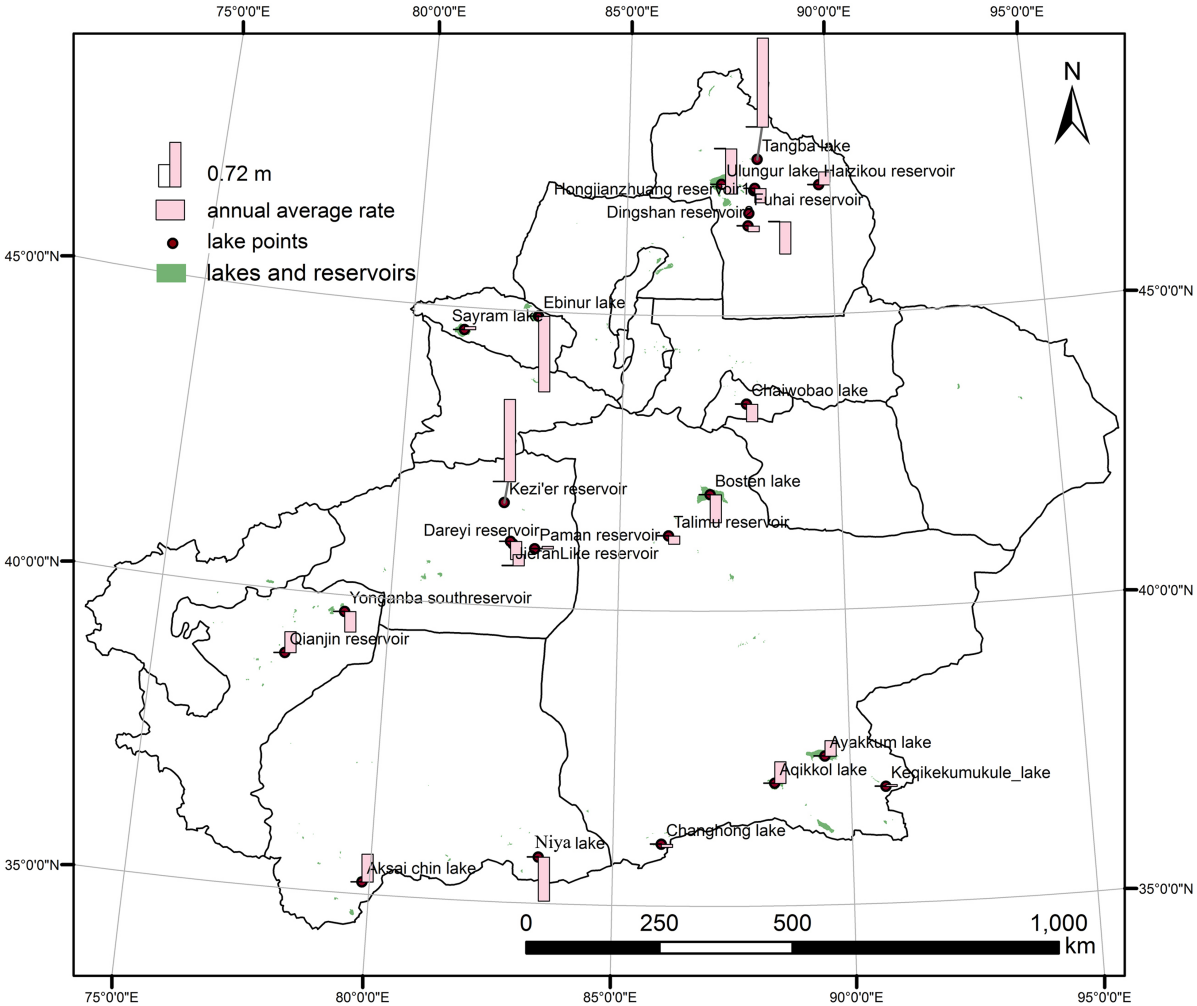
Spatial distribution of annual average rate of water level variation in Xinjiang.

We initially aimed to analyse water level changes over the 56 lakes and reservoirs in Xinjiang that are covered by ICESat/GLAS data. In order to maintain a unified computing standard and make the data more comparable, we finally chose 24 of them with adjacent year data as representatives to analyse the spatial distribution of annual variation rate. According to the geographic coordinates of all of the lakes, the annual average rate of water level exhibits regional characteristics (Fig 7).

From Fig 7, we can see that there are different variation rates among the different landforms of Xinjiang. In the mountains, there are seven lakes, with six located in the south slope of the Kunlun Mountains and one located in the Tianshan Mountains (Figs 1 and 7). Five lakes have an increasing trend, while the remaining two exhibit a decreasing trend. Among the five lakes with increasing trends, there are three lakes with obviously increasing trends (their trends are all greater than 0.25 m per year). Sayram Lake and Keqikekumukule Lake show a small increasing trend (0.05 and 0.03 m per year, respectively). Changhong Lake exhibits a slight decreasing rate (-0.05 m per year), and Niya Lake shows a dramatic decreasing rate (-0.71 m per year).

In the oases, there are four lakes and 12 reservoirs distributed in the middle and north of Xinjiang. Among the four lakes, three show a decreasing trend. Bosten Lake has the most dramatic decreasing rate, at -0.45 m per year. The water level of Bosten Lake decreases from 1048.38 m in 2003 to 1045.67 m in 2009. Tangba Lake has a substantial increase, at +1.44 m per year.

Among the 12 reservoirs, seven exhibit a decreasing trend, with the remaining five showing an increasing trend. Hongjianzhuang Reservoir2 has the greatest decrease, with an annual average rate of -0.73 m. Kezi'er Reservoir presents the most dramatic increase, at 1.33 m per year. The Paman Reservoir has the lowest increasing trend, at 0.04 m per year. Water level variations of reservoirs in the oasis regions are quite different, which is related to their intricate influencing factors.

In the deserts, the annual rate was only able to be calculated for one of the lakes, Ebinur Lake. Ebinur Lake has a dramatic decreasing rate of -1.22 m per year, which is the greatest decreasing rate of all of the lakes and reservoirs in Xinjiang in this study.

### Annual average rates of water levels in different regions

By Eq (1), the variation rate of water level between two adjacent years for every lake and reservoir can be calculated (Table 3). Excluding 0, standard errors (SE) are between 0.01 and 1.48.

**Table 3:** Variation rates of water level between two adjacent years for every lake and reservoir and their standard errors.

Name	Region	2003–2004	2004–2005	2005–2006	2006–2007	2007–2008	2008–2009	Average	SE
Tangba Lake	oasis	1.04			1.84			1.44	0.40
Bosten Lake	oasis	-0.93	-0.32	-0.22	-0.37	-0.33	-0.55	-0.45	0.11
Ulungur Lake	oasis	0.02	0.14	0.28	-0.35	-0.31	-0.15	-0.06	0.10
Chaiwobao Lake	oasis			-0.21	-0.22	-0.40		-0.28	0.06
Sayram Lake	mountain	0.28	0.06	-0.04	0.11	-0.15		0.05	0.07
Aqikkol Lake	mountain	0.45	0.46	0.37	0.34	0.29	0.17	0.35	0.04
Ayakkum Lake	mountain	-0.24	0.92	0.30	-0.15	0.22	0.47	0.25	0.17
Aksai chin Lake	mountain	0.08	0.48	1.25	0.17	0.30		0.45	0.21
Keqikekumukule_Lake	mountain		0.02	0.03				0.03	0.01
Changhong Lake	mountain				0.12	-0.43	0.15	-0.05	0.19
80# Lake	mountain				-0.71			-0.71	0.00
Ebinur Lake	desert	-1.22						-1.22	0.00
Qianjin Reservoir	oasis		0.34					0.34	0.00
Kezi'er Reservoir	oasis			0.77	1.89			1.33	0.56
Paman Reservoir	oasis	-1.29	1.07	0.16	0.15	0.10		0.04	0.38
Dareyi Reservoir	oasis	0.26			0.51			0.38	0.13
Hongjianzhuang Reservoir2	oasis				-0.29	-1.18		-0.73	0.44
Haizikou Reservoir	oasis	0.21						0.21	0.00
Fuhai Reservoir	oasis	1.10	1.98	-2.54	-2.63			-0.52	1.20
Dingshan Reservoir2	oasis	-2.88			2.16	0.44		-0.09	1.48
Yonganba south Reservoir	oasis	-1.00			-0.91	0.91		-0.33	0.62
JieranLike Reservoir	oasis					-0.29		-0.29	0.00
Hongjianzhuang Reservoir1	oasis				-0.23			-0.23	0.00
Talimu Reservoir	reservoir of oasis			-0.28	0.01			-0.13	0.14

**Fig 8:**
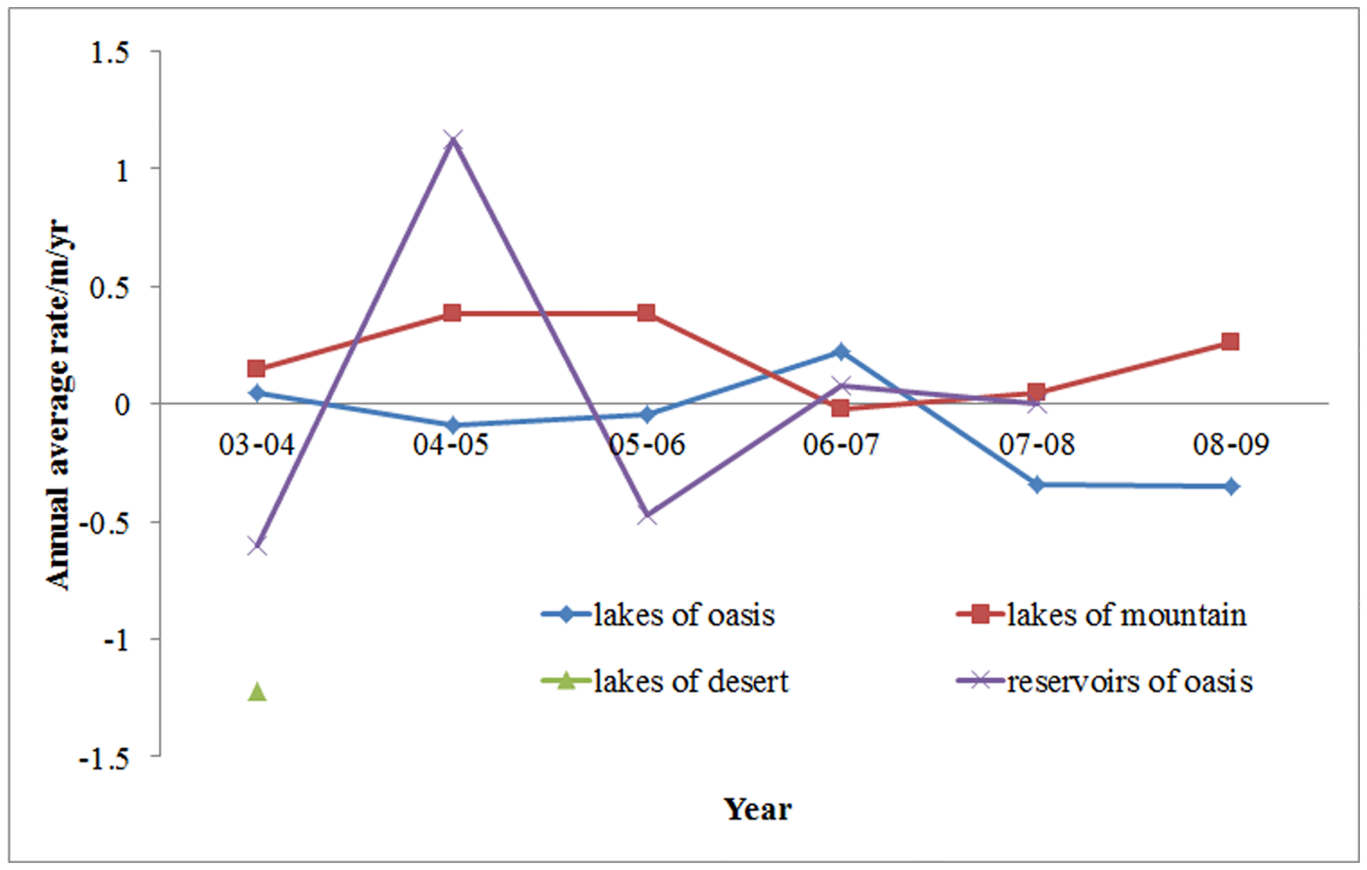
Annual average rates in different geologic environments from 2003–2004 to 2008–2009.

For each two adjacent years, we average all variation rates at the same region (mountain, oasis or desert), and do the same for reservoirs. From Fig 8, for the variation rates of water level in oases, the inter-annual fluctuation of reservoirs is higher, and change between positive and negative values. The smallest is approximately -0.6 m in 2003–2004, while the biggest is approximately 1.13 m in 2004–2005. It is obvious that the water level fluctuation difference of reservoir is very large. In mountainous regions, the water level variation rate of lakes showed a rise-decrease-rise trend, and always had positive values except 2006–2007, which indicates that water levels of lakes in mountainous regions mainly exhibit increases. On the contrary, in oasis regions, the water level variation rate of lakes showed a decrease-rise-decrease trend, and was always negative except 2003–2004 and 2006–2007, which indicates that water levels of lakes in oasis regions are mainly decreasing. Since there is only one lake in the desert regions covered with ICESat/GLAS data and only one variation rate in 2003–2004, we were unable to predict the trend.

### Water volume variation in different regions

The total area of the 50 lakes and reservoirs is 5406.55 km^2^, accounting for 81% of all lakes and reservoirs whose areas are larger than 1 km^2^. Areas of mountainous lakes and reservoirs in these 50 lakes and reservoirs account for 75.5% of the areas of the total mountainous lakes and reservoirs. Similarly, areas of oases and desert lakes and reservoirs account for 82.7% and 93.4%, respectively. According to the water level variation and area of lakes and reservoirs, we can calculate the water volume variation (Eq 2):

ΔV=ΔE×S (2)

where ΔV is the variation of water volume (a positive value means increase and a negative value means decrease); ΔE is the difference between end elevation and start elevation; and S is the average area of lake or reservoir in 2006, which is the middle year of the study period. In order to simplify the calculation process, we assume that the area of lakes changes in a single direction (increase or decrease or remain stable year by year), and there is no mutation. We also assume that *S* in 2006 equals (*S*_*t1*_+*S*_*t2*_)/2, where *S*_*t1*_ and *S*_*t2*_ is the average area of lake or reservoir before and after the changes of water level, respectively.

**Fig 9:**
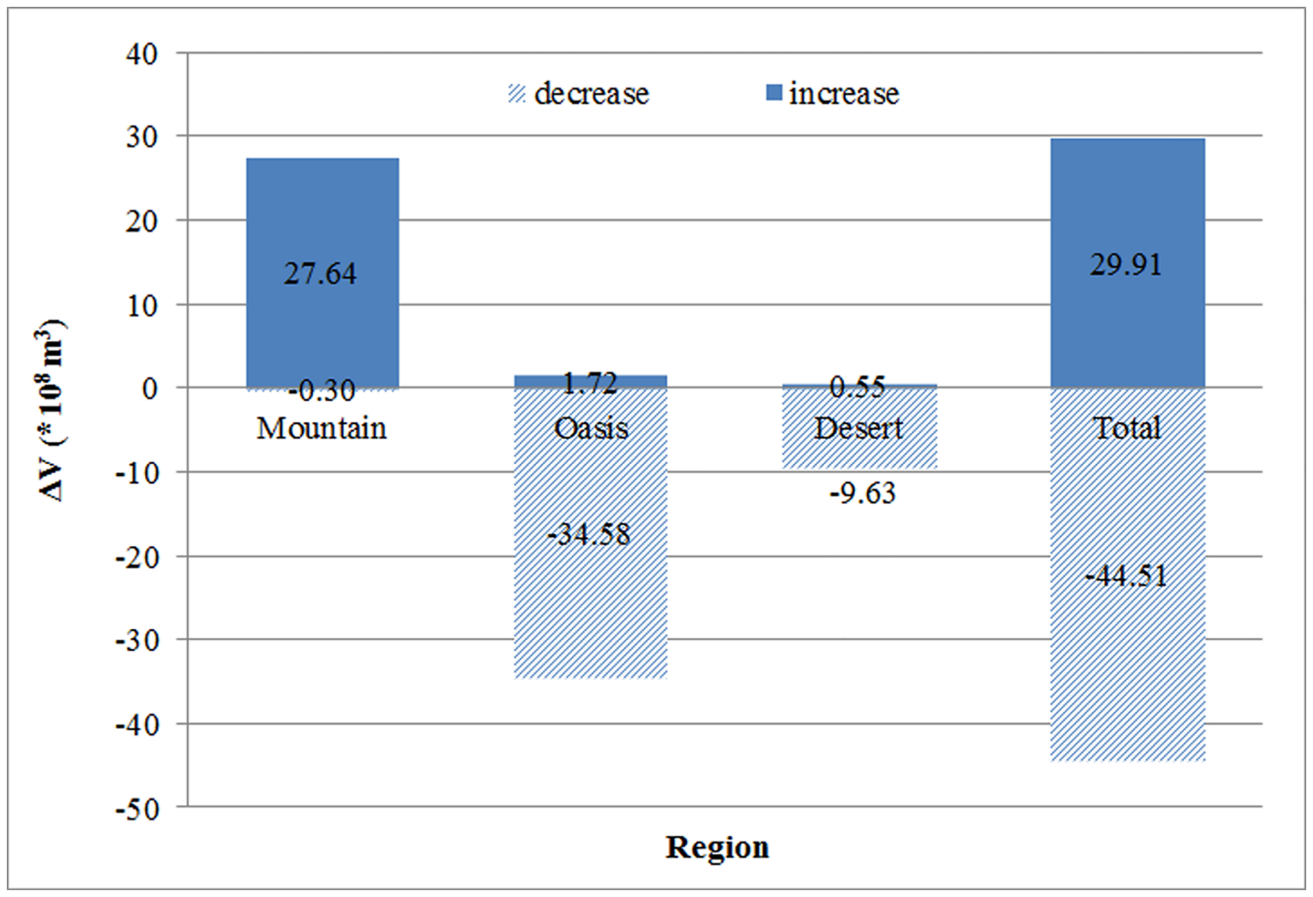
Variation of water volume for lakes and reservoirs in different regions from 2003 to 2009.

ΔVs are shown as S1 Table. We calculated the total ΔV by adding up all increasing volumes and decreasing volumes separately in different regions. As shown in Fig 9, water volume increases are larger than volume decreases in mountainous regions. In oasis regions, water volume decreases are larger than volume increases. Furthermore, water volume decreases in oasis regions are also larger than increases in mountainous regions. In desert regions, water volume is mainly decreasing. Overall, the total increase is less than the amount of reduction. Therefore, considering the change of water level, the water volume of lakes and reservoirs in Xinjiang reduced 14.6×10^8^ m^3^ from 2003 to 2009.

## Discussion

### Reasons for the different variations of water levels in different geologic environments

#### Mountainous regions

Mountainous lakes in Xinjiang are located in the Altay Mountains, Tianshan Mountains, Pamir Mountains and Kunlun Mountains, which were upraised to a height above the snowline. As a result, these mountains are huge glacier centres and become “wetlands” or “greens” of arid zones when receiving abundant precipitation. In addition, the fracture activity intermittently promotes the formation of depressions or mountain basins where melt water convergences, with the damming effects of glaciers, mudslides, collapses, landslides, and other debris. These conditions have created favourable conditions for the birth of mountainous lakes.

The climatic processes affecting glaciers, both modern and those of the past, are unique to high altitudes and/or high latitudes, which are typically areas with few instrumented climate stations [41]. The mountain glaciers in western China have experienced losses of mass and volume over the past several decades [42]. Mountains are “reservoirs” in the arid regions of northwest China that receive a relatively great amount of precipitation. This is also the main region of the distribution of glaciers. There is also high biological diversity in complex ecosystems in mountains.

Most studies have found that the arid regions of northwest China are becoming warmer under the background of global warming. Li et al. [7] studied temperature variations during the snowmelt period, as well as its effect on the runoff in the mountain areas of northwest China from 1960 to 2010.The study revealed that: 1) the mean air temperature and the temperature in northern Kunlun Mountains increased by 0.857°C and 0.617°C, respectively; and 2) temperature changes in the snowmelt period resulted in an annual runoff change of 14.15%, which is greater than that of precipitation. Most of the mountainous lakes are recharged by snow or glacial melts. Therefore, the rising temperature trends will lead to increasing melting of glaciers and snow, subsequently resulting in the rising of water levels in lakes [32]. Glacier runoff is an important water resource in arid northwest China [43].

Most lakes show an obvious increasing trend (Ayakkum Lake, Aksai chin Lake, and Aqikkol Lake). According to the “Records of Lakes in China” [34], Ayakkum Lake, Aksai chin Lake, and Aqikkol Lake are mainly recharged with snow or glacial melts. Basins are snow-covered year round. For example, Aksai chin Lake has only one river flowing into the lake, named the Aksai chin River. The river originated in the peak of the Kunlun Mountains in the northeast with a total glacial area of approximately 709.8 km^2^, ice volume of 136.27 km^3^, and abundant water. Therefore, the warming trend could probably result in more melting and snow, subsequently leading to a rise of water levels in lakes that are recharged with snow or glacial melt. This observation has also been demonstrated in other studies [9, 24, 44, 45].

Although Sayram Lake and Keqikekumukule Lake are both mountainous lakes, melt-water is not the main supply source because there are fewer glaciers and sources of permanent snow in the basin, and there are no large rivers that flow into the lakes. The main water supply sources for Sayram Lake are rain and groundwater [34]. The water source of Keqikekumukule Lake is dependent on the 3 km long river from the southeast that brings two spring recharges. As demonstrated, temperature is not the main influential factor. In mountainous region, the water levels of lakes, which are less affected by human activity, will change with precipitation and evaporation variation.

The decreasing trend of Niya Lake is different from the other lakes. By observing the surrounding terrain, there is no glacier or permanent snow, and no river flowing into the lake. By analysis of original data, there is a large decline in October between 2007 and 2008 and increase in other months. For Changhong Lake, there is the same pattern as Niya Lake. Therefore, in order to determine why the variation rate of the water level of these two lakes is different from other lakes, we need to collect much more surrounding climate, hydrology, snow, and other data during the same period. The reasons for the decreasing trend remain unclear, and further analysis is needed.

#### Oasis regions

Oases are a kind of heterogeneity ecological landscape with relative stability and significant microclimate effect, which is based on a sizeable biological community in a small scale under the substrate of a large-scale desert. Desert ecosystems are the material and ecological base of oases. They require zonal distribution near rivers or wells, springs, and piedmont having ice and snow melt water irrigation. Oases are often places where farming and animal husbandry have developed in arid regions. In Xinjiang, oases occupy less than 8% of the total area, but constitute more than 90% of the population and farmland.

For the lakes in the oasis regions, the water level of Bosten Lake shows the greatest decreasing trend (Figs 3 and 7). This could be due to the following reasons.

The first one is decrease of runoff recharge. Changes of river runoff in the watershed have a direct relationship with changes of the lake water level. Runoff from the Kaidu River accounted for 85% of the total runoff that flowed into Bosten Lake [32]. Therefore, the Kaidu River, as the main water supply of Bosten Lake, had a great impact on the level of Bosten Lake. According to internal data collected from the Xinjiang Tarim River Basin Management Bureau in 2010, runoff of the Kaidu River was 49.68×10^8^ m^3^ in 2000, and reduced to 37.16×10^8^ m^3^ in 2009, decreasing more than 25%. Especially in 2004, runoff reached the lowest level on record, at 33.061×10^8^ m^3^. On the other hand, recharge volume also decreased, from 42.296×10^8^ m^3^ in 2000 to 18.451×10^8^ m^3^ in 2009. A reduction of recharge volume is an important factor for lake shrinkage and water level decrease.

Secondly, irrigation water increasing also makes water levels decrease. The expansion of cultivated land is widespread along with the economic development and population growth of Xinjiang in recent decades. In 2000, the irrigation area of the Kaidu River and Kongque River was 1.001×10^3^ km^2^ and 0.723×10^3^ km^2^, respectively, comprising 1.724×10^3^ km^2^ in total. In 2009, the irrigation area of the rivers increased to 1.098×10^3^ km^2^ and 0.979×10^3^ km^2^, respectively, totalling 2.077×10^3^ km^2^. Although the irrigation area increased by 352.7 km^2^, agricultural irrigation water did not show a significant increase because improvement of water-saving technology decreased the irrigation norm. From 2000 to 2009, irrigation water of the Kaidu River and Kongque River increased from 9.001×10^8^ m^3^ and 8.320×10^8^ m^3^, to 9.951×10^8^ m^3^ and 8.607×10^8^ m^3^, respectively.

**Fig 10:**
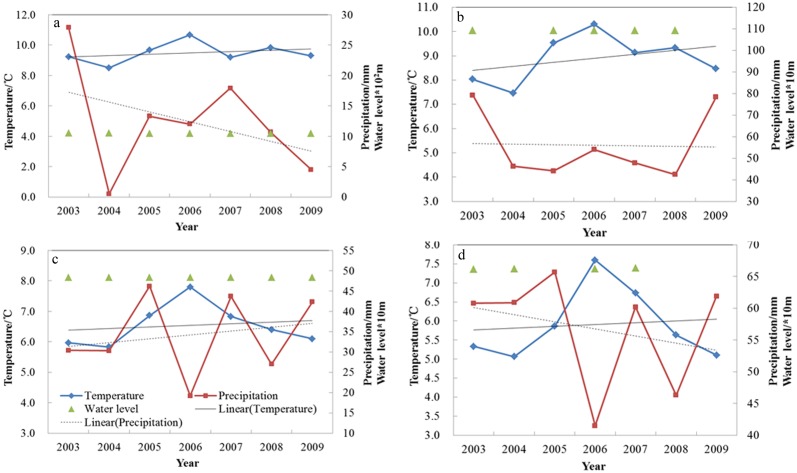
Temperature, precipitation, and water level changes in Bosten Lake (a), Chaiwopu Lake (b), Ulungur Lake (c), and Tangba Lake (d).

Thirdly, the combination of temperature and precipitation affected the change of water levels in lakes. Fig 10 presents the changes of autumn average temperature, precipitation, and water level of Bosten Lake (a), Chaiwopu Lake (b), Ulungur Lake (c) and Tangba Lake (d), respectively, from 2003 to 2009. As Fig 10a shows, temperature exhibits a slightly increasing trend, which can lower water levels due to enhanced evaporation. Precipitation has a decreasing trend, which also contributes to decrease in water level. Both of them result in the decline of water level.

Chaiwopu Lake has the second greatest decreasing trend in annual water level variation. Climatic factors and human activity jointly cause the water level decrease. From the analysis of fall temperature and precipitation data from the Urumqi weather station near Chaiwopu Lake from 2003 to 2009, temperature shows an increasing trend and precipitation shows a fluctuated decreasing trend (Fig 10b). This result is consistent with Ma et al. [28]. Ma et al. [28] analysed temperature and precipitation from the Urumqi and Dabancheng weather stations, and concluded that temperature showed an increasing trend from 1985 to 2009, while basin precipitation decreased in recent years. Strong evaporation caused by high temperature, coupled with a reduction in rainfall, could lead to a decline in water level. Ma et al. [28] further revealed that the decisive influential factor on the water level decline was groundwater over exploitation. To meet the demand of water in Urumqi in 1993 and 1999, water sources of north Chaiwopu and west Chaiwopu were built to extract groundwater, which led to an obvious downward trend of groundwater tables in the water sources. According to Qu et al. [30], groundwater accounted for 90.4% of the total recharge for Chaiwopu Lake. The decrease of groundwater amount into the lake caused the drop of the lake level. Chaiwopu Lake is the largest freshwater lake in Urumqi. With economic development, the population and cultivated land area of Urumqi increases each year. From the end of 2003 to the end of 2008, the population increased from 18.153 million to 23.605 million, a growth of 5.452 million; the cultivated land area increased from 320.9 km^2^ to 969.5 km^2^ [46–47]. A number of new wells were built for emergencies caused by drought, which increased groundwater extraction. The increase of population and cultivated land area increased water consumption around Chaiwopu Lake, which made the levels of supplemental water flowing into the lake decrease, causing the lake level to drop.

Ulungur Lake is located on the northern edge of the Autonomous Xinjiang Province, China. It is fed by the Ulungur River at its southeast margin and has no outflow [26]. The water level of Ulungur Lake decreases slightly. Fig 10c shows that temperature has a slightly increasing trend, and precipitation exhibits an increasing trend. Wu et al. [27] indicated that, due to the climate impact of drought, the water level of Ulungur Lake continued to decline. This trend was obvious from 2005 to 2008.

Regarding Tangba Lake, which has been artificially transformed into a reservoir [48], an increasing temperature trend and decreasing precipitation trend have not made the water level decrease (Fig 10d). The rising trend of the water level of Tangba Lake is greatly influenced by government regulations.

The results demonstrate that lake level variations within the oasis regions are affected by natural factors, combined with human activity.

For 12 reservoirs in the oasis regions, seven exhibited declining trends. From the end of 2003 to the end of 2008, the total population of Xinjiang Province increased from 193.395 million to 213.081 million; the cultivated land area expanded from 33139.9 km^2^ to 41245.6 km^2^ [46–47]. Both of them increased water demand, which directly led the water level of most reservoirs to decrease. The water level of reservoirs is mainly influenced by human activity. For example, the annual discharge gate opening time, changes of local water regulations, the year storage capacity, etc., can all impact the water level of the reservoirs. Taking into account numerous and complex factors, we need to analyse particular issues for each specific reservoir in order to identify the actual influential factors.

#### Desert regions

Deserts are characterized by vast areas, sparse vegetation, and fragile ecosystems. There is only one lake covered by the ICESat/GLAS orbits with adjacent year data. Ebinur Lake is located in the northwest part of Xinjiang Province. The annual average precipitation is only 95 mm and annual average evaporation is as high as 1315 mm [29]. Ma et al. [29] revealed that the surface area of Ebinur Lake reached peaks at 903 km^2^ in 2003, decreased to 847 km^2^ in 2004, and further to 746 km^2^ in 2005. Wang et al. [31] analysed the reasons for the water level variation of Ebinur Lake in the past 50 years. They concluded that, from 1950 to 2001, the surface area went through three stages: rapidly shrinking, stable, and gradually increasing. They further anticipated that Ebinur Lake will enter a dry period in the future, and the runoff flowing into the lake will also decrease. This means a reduction in the water level of the lake. The predicted results coincide with the results of this study.

### Comparison with similar studies

56 of the 100 largest lakes in China were studied by Wang et al. [2], of which, only six (Ayakkum Kul Lake [named as Ayakkum Lake], Aksai Chin Lake, Achik Kul Lake [named as Aqikkol Lake], Bosten Lake, Syram Lake and Buluntuo Lake [named as Ulungur Lake] are located in Xinjiang. In this study, 56 of the 155 lakes (including reservoirs) with areas larger than 1 km^2^ were derived from ICESat/GLAS data during the period of 2003 to 2009. These data are more sufficient for studying general water level variations of lakes and reservoirs in different geologic environments in Xinjiang, and determining why the differences exist. By comparing our results with Wang et al. [2] (Table 4), the increasing or decreasing trend is consistent with each other, but the growth rate values are slightly different. This is due to our different calculation method for annual variation rate of water level. In this paper, in order to avoid the effects of inter-annual changes on annual variation rate, we chose data from late October to early November.

**Table 4:** Comparison with Wang et al. for annual average rate of water level.

Unit: m/yr			
Lakes	Wang et al.	Our results	Difference
Ayakkum Lake	0.251	0.253	-0.002
Aksai chin Lake	0.507	0.455	0.052
Aqikkol Lake	0.395	0.348	0.047
Syram Lake	0.044	0.053	-0.009
Bosten Lake	-0.393	-0.453	0.06
Ulungur Lake	-0.081	-0.060	-0.021

Zhou’s master's thesis [49] introduced the distribution characteristics of lakes and dry salt lakes (areas larger than 1 km^2^), including the number of lakes and the change of their distribution and area. However, he did not analyse water level changes. In terms of area, the result in this study is greater than Zhou [49] and Wang and Dou [34] (Table 5). The main reason for this is that, in this study, the area calculation includes lakes and reservoirs, while Zhou (2009) and Wang and Dou [4] only count for lakes. On this point, this study has a broader scope. Regarding the number of lakes, however, this study is more limited than Zhou [49] and Wang and Dou [4]. The reason for this may be that we treat most reservoirs transformed by lakes as reservoirs.

**Table 5:** Comparison with other results for area.

	Larger than 10 km^2^	1–10 km^2^	Total
Zhou [49]	5972.22	276.00	6248.22
Wang and Dou [34]	5869.59	328.35	6197.94
Our study	6300.92	371.08	6672

Another similar study investigates lake water level variations in the Tibetan Plateau. Zhang et al. [9] divided the Tibetan Plateau into four subareas according to geographical position and the trend of water level variation, which constitutes a good example for our study. Subarea I is east of 94°E; Subarea II is north of 32°N and west of 94°E; Subarea III is between latitude 30°N and 32°N; and Subarea IV is south of 30°N. In view of the special geographic settings and features of Xinjiang, we group lakes and reservoirs into three subareas: mountainous regions, oasis regions and desert regions. This categorization makes the analysis results more reasonable.

## Conclusions

Lakes and reservoirs play an important role in Xinjiang, China’s water supply. Lake water level is particularly sensitive to regional climatic changes. Declining water levels of lakes and reservoirs will produce a series of environmental problems, such as wetland area shrinkage, species diversity reduction, water quality deterioration, fish production reduction, etc. Keeping water levels of lakes at a reasonable level is critical for the purposes of environmental protection. Consequently, it is necessary to understand the status of water levels and variations of lakes and reservoirs in Xinjiang. However, for many lakes, particularly those in remote areas with harsh environmental conditions, there is no *in situ* gauge to measure lake water levels. Although gauge data may exist for some lakes, they are not generally accessible to researchers or to the public. ICESat, launched in January 2003, made it possible to obtain water levels where traditional gauges do not exist. This study indicates that it is feasible to monitor the fluctuation of inland water bodies using ICESat/GLAS data. Comparing the gauge data with ICESat/GLAS data of Bosten Lake, it was demonstrated that a high correlation exists between them (R^2^ = 0.9904), and the systematic difference is 0.15 m. In other words, the more gauge data obtained, the more efficiently the precision of the ICESat/GLAS data can be evaluated.

In terms of seasonal variation, the water levels of lakes in oasis areas are affected mainly by human activity, and water levels in spring are higher than those in autumn. Mountainous lakes are affected by climate changes and glaciers melting, and water levels in spring are lower than those in autumn. There is no specific law for reservoirs in the oasis areas.

In terms of the inter-annual variation, the water levels of lakes distributed in different systems will be affected by varied factors. For different regions, there are different reasons for water level variation. In the mountainous regions, most lakes exhibit an increasing trend and the lakes that are recharged by snow or glacial melting, show an especially increasing trend. This is because they are sensitive to climate warming, which results in increased melting of snow and glaciers. Conversely, the water level change of lakes in mountainous regions will reflect climate changes, and constitute one of the most important indicators of climate changes. In general, natural factors played an important role for the lakes in mountainous areas. Most lakes in the oasis regions exhibit a decreasing trend of water level change, probably due to runoff recharge decrease, precipitation reduction, and temperature increase. People’s living water and irrigation water consumption also contributes to the decrease. Bosten Lake shows the greatest decreasing rate (-0.45 m per year) because of the decrease of runoff recharge and increase of irrigation water. Most reservoirs in the oasis regions also show a declining trend. Human activity is identified as the main influencing factor. In the oasis regions, the annual average rate of lakes is higher than that of reservoirs. The main reasons for the decreasing water levels in the desert regions are the reduction of recharged runoff and high evaporation. These conclusions are similar to Wu et al. [35], whose study demonstrated that mountainous lake changes could be a reliable indication of climate change because these lakes were rarely influenced by human activity. Oasis lake changes, on the other hand, are related to human activity occurring in the basin, as well as the morphological characteristics of lakes and stages of evolution in the lakes. Lakes in desert regions are more dependent on changes of precipitation in mountainous basins.

Analysis of water level and water volume variations in different regions showed an increasing trend in mountainous regions, and a decreasing trend in oasis regions and desert regions. Total water volume exhibits a declining trend. According to data from the “Xinjiang Water Resource Bulletin” [50–51], surface water resources are reduced 149.5×10^8^ m^3^ (from 863.2×10^8^ m^3^ in 2003 to 713.7×10^8^ m^3^ in 2009). Water volume of lakes and reservoirs is reduced 14.6×10^8^ m^3^ from 2003 to 2009, accounting for 10% of the total surface water resource reduction. Studies on the water level and volume of Xinjiang lakes and reservoirs could offer important practical significance. The findings of this study can provide useful guidance for future water resource allocation and regulation in Xinjiang.

## Supporting information

S1 Table Data for 50 lakes and reservoirs in Xinjiang, China. (DOCX)
