# Supplementary material for: Analysis of water level variation of lakes and reservoirs in Xinjiang, China using ICESat laser altimetry data (2003–2009)
Source: PLoS One. 2017 Sep 5;12(9):e0183800. doi: 10.1371/journal.pone.0183800 (PMC5584802; doi:10.1371/journal.pone.0183800)
Supplement: S1 Table — (DOCX) [file pone.0183800.s001.docx]

S1 Table. Data for 50 lakes and reservoirs in Xinjiang, China

| Region | ID | Names | E  (°) | N  (°) | Area  (km^2^) | Campaigns  (years) | Start date | End date | Start elev | End elev | ΔE  (m) | ΔV  (m^3^) |
| --- | --- | --- | --- | --- | --- | --- | --- | --- | --- | --- | --- | --- |
|  |  |  |  |  |  |  | (yymmdd) | | (m) | |  |  |
| Mountain | 4 | **Ayakkum Lake** | 89.4700 | 37.5200 | 776.2650 | 13(7) | 030223 | 090329 | 3879.61 | 3881.24 | 1.63 | 1265311869 |
| Mountain | 48 | **Aqikkol Lake** | 88.3960 | 37.0753 | 435.4033 | 16(7) | 031018 | 091002 | 4255.06 | 4257.14 | 2.08 | 905638951 |
| Mountain | 10 | **Aksai chin Lake** | 79.8570 | 35.2135 | 189.1541 | 15(7) | 031025 | 090317 | 4851.90 | 4854.10 | 2.20 | 416138947 |
| Mountain | 6 | **Sayram Lake** | 81.2200 | 44.6200 | 462.8772 | 12(6) | 031024 | 081012 | 2074.32 | 2074.57 | 0.25 | 115719301 |
| Mountain | 24 | **Changhong Lake** | 86.0113 | 36.0484 | 24.1857 | 13(6) | 040227 | 091011 | 4906.08 | 4908.45 | 2.37 | 57320109 |
| Mountain | 50 | **Keqikekumukule Lake** | 90.7415 | 36.9745 | 17.3289 | 12(5) | 040318 | 081214 | 4104.76 | 4104.98 | 0.22 | 3812351 |
| Mountain | 175 | Lake | 83.4895 | 35.7459 | 1.3743 | 4(4) | 050605 | 060310 | 4908.44 | 4908.56 | 0.12 | 168180 |
| Mountain | 26 | Ixakpatti Lake | 90.2833 | 37.3167 | 13.3047 | 1(1) | 031103 | 031103 | 3889.46 | 3889.46 | 0.00 | 0 |
| Mountain | 159 | Ulug Kel Lake | 81.6239 | 35.6711 | 16.4255 | 1(1) | 051119 | 051119 | 4672.90 | 4672.90 | 0.00 | 0 |
| Mountain | 160 | AkTag Lake | 84.4557 | 36.5891 | 11.4525 | 1(1) | 031011 | 031011 | 4232.65 | 4232.65 | 0.00 | 0 |
| Mountain | 185 | Tso Tang Lake | 79.3324 | 34.8929 | 19.8098 | 1(1) | 040621 | 041107 | 5210.03 | 5209.77 | **-0.26** | **-5094991** |
| Mountain | 80 | **Niya Lake** | 83.4583 | 35.7813 | 21.4461 | 15(7) | 031101 | 090324 | 4905.29 | 4904.76 | **-0.53** | **-11366433** |
| Mountain | 16 | Kanasi Lake | 87.0412 | 48.8148 | 44.0343 | 1(1) | 030223 | 030311 | 1368.25 | 1367.95 | **-0.30** | **-13382574** |
| Oasis | 33 | **Haizikou Reservoir** | 89.7268 | 47.1845 | 19.4467 | 11(5) | 040314 | 081210 | 1155.62 | 1158.34 | 2.72 | 52895146 |
| Oasis | 75 | **Qianjin Reservoir** | 77.8235 | 38.9358 | 20.0400 | 11(5) | 040314 | 081210 | 1170.28 | 1172.10 | 1.82 | 36472861 |
| Oasis | 113 | **Kezi'er Reservoir** | 82.3840 | 41.7453 | 34.9030 | 15(6) | 031101 | 080304 | 1147.77 | 1148.57 | 0.80 | 27922404 |
| Oasis | 23 | **Tangba Lake** | 88.2129 | 47.6372 | 13.2390 | 13(6) | 031027 | 080227 | 661.35 | 663.40 | 2.05 | 27139950 |
| Oasis | 125 | Yuejin Reservoir | 86.2299 | 44.4352 | 13.9842 | 12(7) | 040614 | 090405 | 411.43 | 412.43 | 1.00 | 13942859 |
| Oasis | 154 | **Paman Reservoir** | 83.1142 | 40.9909 | 27.0666 | 12(6) | 031020 | 081007 | 959.60 | 959.88 | 0.28 | 7578640 |
| Oasis | 68 | Honghai Reservoir2 | 78.3529 | 39.7553 | 14.8297 | 2(1) | 030223 | 030927 | 1124.31 | 1124.56 | 0.25 | 3667003 |
| Oasis | 173 | Reservoir | 87.4963 | 44.3253 | 2.3879 | 3(2) | 031027 | 080227 | 452.40 | 452.95 | 0.55 | 1307799 |
| Oasis | 158 | Yapuquan Reservoir | 78.1363 | 37.4337 | 1.4839 | 7(5) | 040314 | 080314 | 1668.72 | 1669.18 | 0.45 | 673443 |
| Oasis | 18 | Salt Lake | 88.1000 | 43.3920 | 10.1303 | 3(2) | 030228 | 040306 | 1072.06 | 1072.08 | 0.02 | 198249 |
| Oasis | 7 | Lake | 87.8869 | 47.4943 | 9.0855 | 1(1) | 040310 | 040310 | 498.17 | 498.17 | 0.00 | 0 |
| Oasis | 9 | Jili Lake | 87.4300 | 46.9200 | 168.2937 | 1(1) | 041108 | 041108 | 483.28 | 483.28 | 0.00 | 0 |
| Oasis | 17 | Ailike Lake | 85.7916 | 45.9373 | 54.9288 | 1(1) | 031014 | 031014 | 277.32 | 277.32 | 0.00 | 0 |
| Oasis | 28 | Lake | 87.5304 | 46.8794 | 13.5891 | 1(1) | 041108 | 041108 | 483.10 | 483.10 | 0.00 | 0 |
| Oasis | 122 | Moguhu Reservoir | 85.9212 | 44.4635 | 14.5296 | 1(1) | 031011 | 031011 | 385.47 | 385.47 | 0.00 | 0 |
| Oasis | 151 | Fengchenggaoku Reservoir | 85.6363 | 46.2150 | 5.0832 | 1(1) | 031003 | 031003 | 467.95 | 467.95 | 0.00 | 0 |
| Oasis | 155 | Ka'erQuga Reservoir | 85.2620 | 40.9450 | 3.0834 | 1(1) | 031011 | 031011 | 909.26 | 909.26 | 0.00 | 0 |
| Oasis | 180 | Dahaizi Reservoir | 86.7687 | 44.4250 | 5.8157 | 1(1) | 060526 | 060526 | 420.99 | 420.99 | 0.00 | 0 |
| Oasis | 54 | Daxihaizi Reservoir | 87.5297 | 40.5738 | 57.0961 | 2(1) | 030324 | 031002 | 850.47 | 850.44 | **-0.03** | **-1701959** |
| Oasis | 156 | **Talimu Reservoir** | 86.0992 | 41.2694 | 12.0843 | 9(6) | 040523 | 090311 | 886.80 | 886.63 | **-0.17** | **-2054331** |
| Oasis | 145 | Ayikule Reservoir | 84.8410 | 45.5579 | 3.6468 | 10(7) | 030304 | 090320 | 362.58 | 361.60 | **-0.98** | **-3556639** |
| Oasis | 140 | **Hongjianzhuang Reservoir1** | 88.1411 | 47.1477 | 8.1198 | 8(4) | 040528 | 071013 | 574.20 | 573.63 | **-0.57** | **-4628286** |
| Oasis | 36 | **Dingshan Reservoir2** | 87.9624 | 46.5159 | 5.1014 | 13(6) | 031027 | 081014 | 611.94 | 610.99 | **-0.95** | **-4846350** |
| Oasis | 161 | **Dareyi Reservoir** | 82.6276 | 41.0526 | 10.6784 | 11(7) | 031114 | 090406 | 970.45 | 969.97 | **-0.48** | **-5125609** |
| Oasis | 116 | **JieranLike Reservoir** | 82.5671 | 41.0950 | 23.8598 | 10(6) | 031101 | 081130 | 972.01 | 971.64 | **-0.37** | **-8828123** |
| Oasis | 34 | **Fuhai Reservoir** | 87.9857 | 46.7263 | 18.9342 | 12(7) | 031027 | 090330 | 573.63 | 572.90 | **-0.73** | **-13821987** |
| Oasis | 21 | **Hongjianzhuang Reservoir2** | 88.1152 | 47.1091 | 8.4204 | 9(6) | 040528 | 081014 | 578.18 | 576.46 | **-1.72** | **-14483088** |
| Oasis | 112 | Xinjingzi Reservoir | 79.8257 | 40.4828 | 13.6865 | 2(1) | 030226 | 030930 | 1056.99 | 1055.88 | **-1.11** | **-15167488** |
| Oasis | 65 | Xiaohaizi Reservoir | 78.7533 | 39.7315 | 107.6368 | 2(2) | 040608 | 050312 | 1115.23 | 1115.02 | **-0.21** | **-22881796** |
| Oasis | 19 | **Chaiwopu Lake** | 87.8800 | 43.5000 | 29.1581 | 15(7) | 031030 | 090322 | 1092.97 | 1092.05 | **-0.92** | **-26825456** |
| Oasis | 103 | **Yonganba south Reservoir** | 79.0292 | 39.7190 | 49.3050 | 15(7) | 031025 | 090317 | 1102.62 | 1101.93 | **-0.69** | **-34020457** |
| Oasis | 42 | Shengli Reservoir | 81.0656 | 40.4757 | 46.3766 | 4(3) | 031016 | 050520 | 1022.13 | 1021.10 | **-1.03** | **-47676643** |
| Oasis | 31 | **Ulungur Lake** | 87.3200 | 47.2200 | 858.8414 | 18(7) | 030303 | 091003 | 483.65 | 482.89 | **-0.76** | **-652719494** |
| Oasis | 1 | **Bosten Lake** | 87.0330 | 41.9700 | 959.1651 | 17(7) | 031027 | 091011 | 1048.38 | 1045.67 | **-2.71** | **-2599337421** |
| Desert | 3 | Manas Lake | 85.8500 | 45.8000 | 167.0193 | 13(7) | 031014 | 090408 | 250.28 | 250.60 | 0.31 | 52338372 |
| Desert | 167 | Xiaoailike Lake | 85.5765 | 45.7641 | 2.3454 | 2(1) | 030301 | 031003 | 263.96 | 265.20 | 1.24 | 2900521 |
| Desert | 5 | **Ebinur Lake** | 82.9500 | 44.9200 | 560.0944 | 14(7) | 031012 | 090329 | 195.41 | 193.69 | **-1.72** | **-963362373** |

Note: Names in bold show the lakes and reservoirs for inter-annual variation analysis. ΔE and ΔV in bold show the lakes and reservoirs with the decrease of water level and reduction of water volume.
